# Supplementary material for: Screening the pandemic response box identified benzimidazole carbamates, Olorofim and ravuconazole as promising drug candidates for the treatment of eumycetoma
Source: PLoS Negl Trop Dis. 2022 Feb 4;16(2):e0010159. doi: 10.1371/journal.pntd.0010159 (PMC8815882; doi:10.1371/journal.pntd.0010159)
Supplement: Attachment — Submitted filename: Reviewer comments Reply Letter PLOSNTD.docx [file pntd.0010159.s002.docx]

**Reviewer's Responses to Questions**

Dear PLoSNTD and reviewers,

I thank you for your time and effort spent in reviewing this manuscript titled ‘Screening the Pandemic Response Box identified Benzimidazole carbamates, Olorofim and Ravuconazole as promising drug candidates for the treatment of eumycetoma.’

In this document we have addressed the issues brought up by reviewers and provided answers accordingly.

We have tried our outmost best in answering the questions raised, you may find the answers below.

Sincerely,

Wilson Lim

(First author and on behalf of all authors)

Reviewer's Responses to Questions

Key Review Criteria Required for Acceptance?

As you describe the new analyses required for acceptance, please consider the following:

----------------------------------------------------------------------------------------------------------------------------------------------

**Methods**

-Are the objectives of the study clearly articulated with a clear testable hypothesis stated?

-Is the study design appropriate to address the stated objectives?

-Is the population clearly described and appropriate for the hypothesis being tested?

-Is the sample size sufficient to ensure adequate power to address the hypothesis being tested?

-Were correct statistical analysis used to support conclusions?

-Are there concerns about ethical or regulatory requirements being met?

**Reviewer #1**:

This work deals with the study of the susceptibility of several eumycetoma causative agents to a group of diverse antimicrobial substances.

-Are the objectives of the study clearly articulated with a clear testable hypothesis stated?

I consider the objectives are very clear, to determine the anti-fungal activity of a series of substances, both, in vitro and in vivo

We thank the reviewer, there were no actions necessary here

-Is the study design appropriate to address the stated objectives?

The main problem is that the authors use their own methods instead of similar standardized methods already available to test fungal isolates. I think that makes reproducibility more difficult.

The *in vitro* susceptibility method used in this study is a standardized *in vitro* susceptibility assay based on the CLSI reference standard M38-A2. Our method only differs in two respects. The first is the inoculum preparation. Mycetoma causative agents often don’t sporulate when they are obtained from patients. In fact, for *Madurella* species the hall-mark of identification is that it forms non-sporulating hyphae. Therefore, we cannot use conidia or spores as a starting inoculum as recommended by the CLSI reference standard M38-A2. In 2002, we developed our sonication method to generate hyphal fragments. Using this method, a standardized hyphal inoculum is obtained. Using *Aspergillus fumigatus* we also demonstrated that an inoculum produced via this sonication method results in the same MICs for amphotericin B and the azoles as an inoculum prepared via the standard M38-A2 method. The other deviation is the use of the viability dye XTT. Since we start with a hyphal inoculum which is turbid at start, we need to use the viability dye to assess the minimal inhibitory concentration. Since the development in 2002, this method has become the standard method for *in vitro* susceptibility testing for eumycetoma causative agents.

We realised it was not clear how the screenings were exactly performed, we therefore added the following to the materials and methods section: “To determine which of the compounds present in the Pandemic Response Box were able to inhibit *M. mycetomatis, M. pseudomycetomatis, M. tropicana, F. senegalensis* or *M. romeroi* growth, the CLSI M38-A2 based *in vitro* susceptibility assay for eumycetoma causative agents was used. In the eumycetoma causative agents optimized protocol. In short, fungi were cultured for 10 days at 37°C in RPMI 1640 medium supplemented with L-glutamine (0.3 g/litre) and 20 mM morpholinepropanesulfonic acid (MOPS). The mycelia were harvested by a 5-min centrifugation and were washed with sterile saline. To homogenize the inoculum, the mycelia were sonicated for 20 s at 28 μm (Soniprep, Beun de Ronde, The Netherlands). The final inoculum was prepared from the homogenized fungal suspension mixed with RPMI medium to obtain a transmission of 70% at 660 nm (Novaspec II; Pharmacia Biotech).”

-Is the population clearly described and appropriate for the hypothesis being tested?

The objectives are clearly described, but I think the methodology used is not enough to get conclusions. For instance, they use only 10 isolates of M. mycetomatis to determine the MIC50, that I consider a low amount, insufficient to get conclusions

For most fungal species 10 isolates is indeed a very low number. However, although we hold one of the largest *M. mycetomatis* collections in the world, we can perform in vitro susceptibility testing on 40 different isolates. The 10 isolates used in this study were selected from those 40 to represent the geographical and genomic diversity of all the isolates we possess. Our aim was to determine if the compounds identified in the IC50 screening would not only inhibit the screening isolate but also other isolates with a different genetic background. That is the reason why we performed the MIC50 testing on 10 isolates. The individual MICs are found in the table present on Github. To show that we only included 10 isolates we added that in the by-line of the table.

-Is the sample size sufficient to ensure adequate power to address the hypothesis being tested?

I do not think so

Most drug discovery projects, like the one we performed here, use only a single isolate of the species under investigation. In this project we confirmed the activity of the most potent compounds in 9 other genetically diverse *M. mycetomatis* isolates. In most other drug discovery projects performed on MMV boxes, this extra step is not taken. The most promising compounds having a low IC50 and activity against multiple isolates were then selected for *in vivo* testing in our *Galleria mellonella* model. For each survival line 45 larvae were used. Therefore, in our opinion, the sample size used to demonstrate which compounds are most potent (*in vitro* and *in vivo*) was enough to come to this conclusion. Reviewers 2 and 3 also recognized that the sample size was sufficient to ensure adequate power to address the hypothesis tested.

-Were correct statistical analysis used to support conclusions?

NA

-Are there concerns about ethical or regulatory requirements being met?

No

**Reviewer #2:**

1. The manuscript under peer-review entitled, “Screening the pandemic response box identified benzimidazole carbamates, olorofim and ravuconazole as promising drug candidates for the treatment of eumycetoma.” deals with search of newer therapeutic modalities in the treatment of one of the significant neglected tropical diseases.

2. This is extensively and well-written manuscript dealing with details of newer antifungals and that too through an innovative programme i.e. Open Source Mycetoma initiative (MycetOS).

3. The subject matter has been nicely introduced, which deals with the importance of eumycetoma vis-a-vis availability of antifungal in the tropical countries, which are usually found to be the developing countries.

4. The Open Source Mycetoma initiative (MycetOS), a drug discovery program to discover new drug candidates was started in the year 2018 and all associated data are made available in an online database. This fills the drug discovery pipeline for the treatment of eumycetoma. They had screened 400 compounds from the MMV Pandemic Response Box in search of those active compounds against causative agents of black-grain eumycetoma. The drugs mentioned in the manuscript (benzimidazole carbamates, olorofim and ravuconazole) are in its continuity as a unique programme. Moreover, fenbendazole was also able to inhibit all five most frequently causative agents of eumycetoma.

We thank reviewer 2 for the summary, there were no actions needed based on these comments

**Reviewer #3: Methods**

-Are the objectives of the study clearly articulated with a clear testable hypothesis stated?

Yes

-Is the study design appropriate to address the stated objectives?

Yes

-Is the population clearly described and appropriate for the hypothesis being tested?

Yes

-Is the sample size sufficient to ensure adequate power to address the hypothesis being tested?

Not applicable

-Were correct statistical analysis used to support conclusions?

Yes

-Are there concerns about ethical or regulatory requirements being met?

No

We thank reviewer 3 for the summary, there were no actions needed based on these comments.

----------------------------------------------------------------------------------------------------------------------------------------------

**Results**

-Does the analysis presented match the analysis plan?

-Are the results clearly and completely presented?

-Are the figures (Tables, Images) of sufficient quality for clarity?

**Reviewer #1:**

-Does the analysis presented match the analysis plan?

Not completely

-Are the results clearly and completely presented?

They are consfusing

-Are the figures (Tables, Images) of sufficient quality for clarity?

Most of them

Table 2 and 3 are now fully visible on the PDF file.

**Reviewer #2:**

5. The percentage growth of causative fungi at different levels like 100 μM and 25 μM, IC50 and MIC50 values, Galleria mellonella larvae survival and in vivo significance of the antifungal from the Pathogen Box, Stasis Box and Pandemic Response Box tested, are well depicted in the text description as well as tabulated form of the manuscript.

6. The details of the Results are given minutely in detail. A total of 400 diverse-drug like compounds from the Pandemic Response box were tested in vitro to determine their potency against M. mycetomatis, M. pseudomycetomatis, M. tropicana, F. senegalensis and M. romeroi. Out of these 400 compounds screened at the concentration of 25 μM, 47, 42 and 37 compounds were able to inhibit the growth of the Sordariomycetes M. mycetomatis, M. pseudomycetomatis and M. tropicana, respectively. The Dendrogram of the causative fungi and other depiction including the chemical formula of some of the drugs are also nicely shown.

**Reviewer #3:** Results

-Does the analysis presented match the analysis plan?

Yes

-Are the results clearly and completely presented?

Yes

-Are the figures (Tables, Images) of sufficient quality for clarity?

Yes, except for table 3 which is only partially visible in the PDF file.

Table 2 and 3 has now been made fully visible in the PDF file.

----------------------------------------------------------------------------------------------------------------------------------------------

**Conclusions**

-Are the conclusions supported by the data presented?

-Are the limitations of analysis clearly described?

-Do the authors discuss how these data can be helpful to advance our understanding of the topic under study?

-Is public health relevance addressed?

**Reviewer #1:**

-Are the conclusions supported by the data presented?

No

The aim of the study was to discover new compounds able to inhibit eumycetoma causative agents as written down in the introduction. By showing the *in vitro* susceptibility results and the *in vivo* efficacy results in our *Galleria mellonella* model we demonstrated that the benzimidazole carbamates fenbendazole and carbendazim, the 8-aminoquinolone derivative tafenoquine and MMV1578570 were able to inhibit all 5 eumycetoma causative agents tested *in vitro*. Fenbendazole, MMV1782387, ravuconazole and olorofim were able to significantly prolong survival in *M. mycetomatis* infected *Galleria mellonella* larvae compared to larvae treated with solvent only. In our opinion this conclusion was supported by the data presented.

-Are the limitations of analysis clearly described?

No

We added the following limitation to the discussion: “One limitation of this study is that the invertebrate *G. mellonella* is genetically seen far apart from human and this can cause differences in therapeutic outcome due to difference in immune system and toxicity. *G. mellonella,* like other invertebrates, only has an innate immune response and lacks the adaptive immune response. This can cause differences in therapeutic outcome of certain drugs. For instance, when two structurally unrelated Hsp90 inhibitors were tested in a *Candida albicans* infection model in *G. mellonella,* no toxicity was noted. However, when the same drugs were tested in a mammalian infection model significant toxicity was noted and the therapeutic efficacy of the compound was lost (REF PMID: 19196973). Therefore, the next step would be to test the compounds which showed activity against the *M. mycetomatis* grain formed in *G. mellonella* in mammalian models in the future”.

-Do the authors discuss how these data can be helpful to advance our understanding of the topic under study?

Yes, they do

-Is public health relevance addressed?

Yes

**Reviewer #2:**

7. The Discussion is very elaborative as well as exhaustive. The findings of the manuscript in the light of contemporary studies have been nicely described. This shows from the pandemic response box that newer antifungals are also equally promising.

8. The References are more than sufficient in number and doing justice with the citations vis-a-vis the finding of the manuscript. The authors have cited almost all the published papers on the issue under question i.e. the oldest to the latest ones.

We thank reviewer 2 for his/her comments. No actions were needed on these comments

**Reviewer #3:** Conclusions

-Are the conclusions supported by the data presented?

Yes

-Are the limitations of analysis clearly described?

Please include a limitation section in the discussion (potential variable response in humans vs larvae model, toxicity of the agents in humans, etc..).

Reviewer 3 suggested to include a limitation section in the discussion.

We added the following: “One limitation of this study is that the invertebrate *G. mellonella* is genetically seen far apart from human and this can cause differences in therapeutic outcome due to difference in immune system and toxicity. *G. mellonella,* like other invertebrates, only has an innate immune response and lacks the adaptive immune response. This can cause differences in therapeutic outcome of certain drugs. For instance, when two structurally unrelated Hsp90 inhibitors were tested in a *Candida albicans* infection model in *G. mellonella,* no toxicity was noted. However, when the same drugs were tested in a mammalian infection model significant toxicity was noted and the therapeutic efficacy of the compound was lost (REF PMID: 19196973). Therefore, the next step would be to test the compounds which showed activity against the *M. mycetomatis* grain formed in *G. mellonella* in mammalian models in the future”.

-Do the authors discuss how these data can be helpful to advance our understanding of the topic under study?

Yes

-Is public health relevance addressed?

Yes

----------------------------------------------------------------------------------------------------------------------------------------------

**Editorial and Data Presentation Modifications?**

Use this section for editorial suggestions as well as relatively minor modifications of existing data that would enhance clarity. If the only modifications needed are minor and/or editorial, you may wish to recommend “Minor Revision” or “Accept”.

**Reviewer #1**: The present work deals with the analysis of susceptibility of a series of compounds against eumycetoma agents. My comments are the following:

1. Change the title, use a more direct phrase, such as “ Studies of susceptibility of…”

In our vision, our manuscript did not only analyse the susceptibility of a series of compounds against eumycetoma agents. We started out by screening the Pandemic Response Box to identify compounds which would be able to inhibit the different mycetoma causative agents and then further triage those compounds to test their *in vivo* potency in order to identify drugs which would not only have activity *in vitro* but also in vivo against the mycetoma grain. Therefore, we feel that the current title which includes the phrase “screening the pandemic box” to indicate the screening pipeline we used and “identified Benzimidazole carbamates, olorofim and ravuconazole as promising drug candidates” to name the compounds we finally identified is appropriate for our study.

2. Pgae 3. Line 81: Change “antibiotics” for “antimicrobials”, and add a reference covering this aspect

The word antibiotics has been changed to antimicrobials. Reference was also added.

3. Page 4, line 104: Why do you used molar concentrations?

Molar concentrations were used because it is the original unit used by MMV in their Open Source Boxes – which the Pandemic Response Box is a part of. Maintaining the molar unit in our manuscript enables easier data sharing across laboratories. Since their release, the MMV boxes have been screened against many different pathogens, by sticking to the molar unit in which the box is provided it makes it easier to compare our results with that of other laboratories.

4. Page 4, line 122: Eliminate fig 1, it has nothing to do with the study

Figure 1 shows the relationship between the Sordariales (*Madurella mycetomatis, Madurella fahalii* and *Madurella pseudomycetomatis*) and the Pleosporaleans (*Falciformispora senegalensis* and *Medicopsis romeroi*). These fungi can all cause eumycetoma and have different antifungal susceptibilities. It is helpful to visually present this difference in lineage to our readers when most may not be mycologists or familiar with these fungi.

5. Page 5, line 139: Ten isolates are not enough to determine the MIC50 value. You may need at least 30

See the comments in the first section. The goal was not to determine the MIC50 data on its own. We screened multiple isolates to determine if the compound was able to inhibit all genetically diverse isolates. We are aware that when a single drug is tested often a higher number of strains is used, however, due to the limited number of isolates available in the world, the labour intensiveness of the assay and the fact that we tested the 9 additional strains merely to determine if the compound was able to inhibit a genetically diverse subset of *M. mycetomatis* isolates, we feel that testing more isolates would not have changed our selection of compounds for the *in vivo* model.

We acknowledge that 10 strains is a limited number and that there might be questions and concerns by readers, therefore to make them aware that the MIC50 was only determined against a limited but a genetically and geographically diverse subset of strains we added this to the by-line of the table. To minimize bias, the 10 isolates used in this publication was carefully selected to represent the geographical and genomic difference of all the isolates we possess.

6. Page 5, line 160: Describe briefly the technique, including: age of culture, count of UFC, medium, etc.

The following few sentences briefly describing the *in vitro* susceptibility methods was added in line 160.

Fungi was cultured for 10 days at 37°C in RPMI 1640 medium supplemented with l-glutamine (0.3 g/litre) and 20 mM morpholinepropanesulfonic acid (MOPS). The mycelia were harvested by a 5-min centrifugation and were washed with sterile saline. To homogenize the inoculum, the mycelia were sonicated for 20 s at 28 μm (Soniprep, Beun de Ronde, The Netherlands). The final inoculum was prepared from the homogenized fungal suspension mixed with RPMI medium to obtain a transmission of 70% at 660 nm (Novaspec II; Pharmacia Biotech).

7. Page 5, line 161: Please refer the CLSI method that you are using to determine the susceptibilities

We added a reference to the CLSI method and also added the parts in which we have to deviate from this method.

8. Please use micrograms per ml throughout the document

See above comment. The authors have decided to continue the usage of the molar unit originally given by MMV to all its Open Access Boxes. This ensures uniformity and easy inter-laboratory data sharing and comparison for this is an open source drug discovery program. All publications on the MMV open source boxes use the molar unit.

Page 6, line 187: What sonifier was used? How did that affect viability? How large were the mycelial fragments?

A sonicator from Soniprep, Beun de Ronde, the Netherlands was used. Sonication is required to ensure mycelial fragments are small enough to avoid needle clogging. Hyphal fragments obtained after sonication are below 40µM and a viability of 2.5% is expected.

Page 6, line 188: Four mg in what volume? Of water? It is too much inoculum, how could you inject it into the larvae without clogging the needle?

Four mg in 40µL phosphate-buffered saline (PBS). In our experience, and due to the non-sporulating nature of these fungi, this amount of inoculum is required for our infection model. When we developed the model we also tested 0.4 mg and 0.04 mg. At these amounts the majority of larvae survived. Only at 4 mg /larvae that the larvae die from infection. To ensure that the model resembles the grain formation in human we used histology to monitor grain formation over time. With histology we demonstrated that using this inoculum grains are formed as early as 4 hours post infection and at 3 days post infections the grains were mature and resembled those seen in human patients.

In order to make the inoculum for the larvae we sonicate the hyphae in the exact same manner as used in mice model. The fragments generated by this method are small enough and homogeneous enough to pass through the needle we use to infect the larvae and we have yet to experience clogging of the needle. This model was developed in 2015 and has been used in multiple studies in eumycetoma.

**Reviewer #2:**

9. Therefore keeping in view of the above-mentioned descriptions, the manuscript may be accepted for publication in our esteem journal.

**Reviewer #3**: (No Response)

----------------------------------------------------------------------------------------------------------------------------------------------

**Summary and General Comments**

Use this section to provide overall comments, discuss strengths/weaknesses of the study, novelty, significance, general execution and scholarship. You may also include additional comments for the author, including concerns about dual publication, research ethics, or publication ethics. If requesting major revision, please articulate the new experiments that are needed.

**Reviewer #1:** It is an interesting work, however the methods used I think are not the adequate. There are many small technical details that the authors need to clarify.

**Reviewer #2**: 10. This is nicely conducted study and consequently well-written manuscript with novel ideas.

**Reviewer #3**: The authors report on the evaluation of 400 drug-like molecules against several black grain mycetoma agents identifying several with activity against them. The effort is outstanding and relevant as it may pave the way for new mycetoma treatments in the near future. The article is very well-written and interesting. I only have a few queries and suggestions.

Please remove sentence “No new classes of antifungal agents with new modes of action are identified or evaluated.” from the author summary for being reiterative.

Sentence was removed. Thank you for the suggestion.

Please correct sentence “In Senegal, patients were given 88 terbinafine 500 mg twice daily for 24 – 48 weeks combined with surgery.” for clarity to “In a report from Senegal,..”

Sentence was corrected to “In a report from Senegal, patients were given terbinafine 500 mg twice daily for 24 – 48 weeks combined with surgery.”

In table 2 Amorolfine is classified as an “Antifungals (agrochemical)”. Nonetheless, it is also employed as a topical antifungal in humans. Please modify the table to include this and specify which of the antifungals labeled as “Antifungals (humans)” use are employed systemically and which topically.

Information for amorolfine in table 2 was corrected as suggested. Route of administration for drugs used on humans was also added into table 2 as suggested.

Do the authors know the reason why ravuconazole clinical studies were discontinued in 2007? If so, please state why.

Ravuconazole was discontinued due to bioavailability issues. The following sentence “clinical studies on ravuconazole have been discontinued in 2007 due to bioavailability issues.” was added into line 335. Appropriate referencing was also added.

Table 3 is only partially visible in the PDF file.

Table 2 and 3 has now been made fully visible in the PDF file.

----------------------------------------------------------------------------------------------------------------------------------------------

Reviewer #1: No

Reviewer #2: No

Reviewer #3: Yes: JA Cardenas-de la Garza
